# Relationship between *LAPTM4B* Gene Polymorphism and Prognosis of Patients following Tumor Resection for Colorectal and Esophageal Cancers

**DOI:** 10.1371/journal.pone.0158715

**Published:** 2016-07-08

**Authors:** Xiaojing Cheng, Xiuyun Tian, Xiaojiang Wu, Xiaofang Xing, Hong Du, Chunlian Zhou, Qingyun Zhang, Chunyi Hao, Xianzi Wen, Jiafu Ji

**Affiliations:** 1 Key laboratory of Carcinogenesis and Translational Research (Ministry of Education/Beijing), Division of Gastrointestinal Cancer Translational Research Laboratory, Peking University Cancer Hospital & Institute, Beijing, China; 2 Key laboratory of Carcinogenesis and Translational Research (Ministry of Education/Beijing), Department of Hepato-Pancreato-Biliary Surgery, Peking University Cancer Hospital & Institute, Beijing, China; 3 Department of Gastrointestinal Surgery, Peking University Cancer Hospital & Institute, Beijing, China; 4 Department of Nosocomial Infection Prevention and Control, Beijing Friendship Hospital, Capital Medical University, Beijing, China; 5 Department of Clinical Laboratory, Peking University Cancer Hospital & Institute, Beijing, China; Peking Union Medical College, CHINA

## Abstract

**Background:**

Lysosome-associated transmembrane-4 beta (*LAPTM4B*) is an oncogene that participates tumorgenesis in a variety of human solid tumors, and it has two alleles named as LAPTM4B*1 and *2. The present study aimed to identify the association of *LAPTM4B* genotype with clinicopathological features and prognosis in colorectal and esophageal cancer patients.

**Method:**

Genotypes of *LAPTM4B* were determined by PCR in 167 colon cancer cases (72 patients in a discovery cohort and 95 patients in a testing cohort), 160 rectal cancer cases and 164 esophageal cancer cases. Association between the *LAPTM4B* gene polymorphism and clinicopathological variables was calculated by Chi-square test or Fisher’s exact test. Patient survival differences were calculated by the Kaplan-Meier method. Prognostic factors were determined with Log-rank test and Cox regression model.

**Results:**

LAPTM4B *1/1 was more frequently detected in colon cancer patients with lymph node metastasis and TNM III+IV stages in total colon cancer (discovery + testing cohorts). LAPTM4B *2/2 decreased in recurrent patients in total colon cancer patients (*P* = 0.045). Kaplan-Meier survival curves and Log-rank test showed that LAPTM4B*1 was correlated with shorter overall survival (OS) in discovery and testing cohorts of colon cancer (*P* = 0.0254 and 0.0292, respectively), but not in rectal and esophageal cancer cases (*P* = 0.7669 and 0.9356, respectively). Multivariate analysis showed that *LAPTM4B* genotype was an independent prognostic factor for OS in total colon cancer [*P* = 0.004, hazard ratio (HR) = 0.432; 95% confidence interval (CI) = 0.243–0.768], but not in rectal and esophageal cancers (*P* = 0.791, HR = 1.073, 95% CI = 0.638–1.804 and 0.998, HR = 1.000, 95% CI = 0.663–1.530, respectively).

**Conclusion:**

These findings suggested that *LAPTM4B* allele *1 was a risk factor associated with poor prognosis in patients with colon cancer, but not in patients with rectal or esophageal cancers. *LAPTM4B* genotype status might be a useful prognostic indicator for patients that need surgical operation in colon cancer.

## Introduction

Colorectal and esophageal cancers are common malignant digestive diseases with high incidence and mortality worldwide [1–3]. Nearly one-half patients are diagnosed at an advanced stage, and there are still lack effective targeted therapies in colorectal and esophageal cancers, so survival rates in these cancers have not been improved markedly compared with hematopoietic and lymphoid malignancies. However, it is beneficial to monitor cancer progression for cancer patients [4].

Lysosomal-associated protein transmembrane-4 beta (LAPTM4B) is an oncogene that is upregulated in various solid cancers [5, 6] and associated with poor prognosis, such as gastric cancer [7–9], hepatocellular cancer [10], lung cancer [11, 12] and [13] and etc. *LAPTM4B* exists as two allelic genes, which have the same sequence except for one 19 bp segment for LAPTM4B *1 and two tight tandem segments for LAPTM4B *2 in the 5’ untranslated region of exon 1 [14]. Previous studies have demonstrated that LAPTM4B *2 allele was associated with significantly elevated risk of cancers, such as lung [15, 16], breast [17, 18], gastric [19], colon [20], ovarian [21], gallbladder cancer [22] and etc. Recent studies also suggested that LAPTM4B *2 was an independent prognostic biomarker for hepatocellular carcinoma [23], lung [24], breast [25], endometrial cancer patients [26] and etc.

According to our previous report, the LAPTM4B*2 allele frequency was 33.2% in colon cancer group, 25.5% in rectal cancer group, 22.7% in esophageal cancer group and 24.1% in health control group, indicating that LAPTM4B*2 was correlated with increased risk of colon cancer (*P* = 0.0016), but not with that of rectal and esophageal cancers [20]. However, there was no report about the association between the existence of two variant alleles of LAPTM4B with the prognosis in patients with colorectal and esophageal cancers. The present study aimed to investigate whether there is a correlation of *LAPTM4B* gene polymorphism with prognosis in colorectal and esophageal cancer patients after surgical resection.

## Materials and Methods

### Patients and Controls

In this retrospective study, we collected 167 colon cancer cases (a discovery cohort including 72 patients from Department of Gastrointestinal Surgery between 1999 and 2006, and a testing cohort including 95 patients from Department of Clinical Laboratory between 1997 and 2006), 160 rectal cancer cases and 164 esophageal cancer cases who were hospitalized in Beijing Cancer Hospital, Peking University School of Oncology between June 1997 and December 2006. All patients underwent surgical resection and were finally confirmed according to the World Health Organization classification. The blood samples were stored in the biological tissue bank of Peking University Cancer Hospital & Institute. The tumor-node-metastasis (TNM) stage was determined according to the classification of the American Joint Committee on Cancer and International Union against Cancer. All patients who participated in our study underwent tumor resection at Clinical Oncology of Peking University with follow-up time of 1 to 209.2 months (median: 54.0 months) for colon cancer, 1–134.5 months (median: 60.0 months) for rectal cancer and 1–123.5 months (median: 37.5 months) for esophageal cancer. At the end of follow up, 56.9% (95/167), 55.3% (84/152) and 74.6% (97/130) patients died from colon, rectal and esophageal cancers, respectively.

This study had been approved by the Research and Ethical Committee of Peking University School of Oncology. Written informed consent was obtained from each patient participated in this study.

### DNA extraction and PCR analysis

Total genomic DNA was isolated from peripheral white cells using Blood Genomic DNA extraction kit following the manufacturer’s instructions (Tiangen Beijing, China). DNA was dissolved in elution buffer, and its concentration was measured with a Nanodrop 2000 spectrophotomer (Thermo Fisher Scientific, Wilmington, Delaware, USA) and stored at -80°C until use.

Genotype of LAPTM4B was identified by PCR analysis using the primers 5’-GCCGACTAGGGGACTGGCGGA-3’ (sense) and 5’-CGAGAGCTCCGAGCTTCTGCC-3’ (antisense). PCR was carried out on a thermo cycler (Gene Cycler TM, Bio-Rad, CA, USA) in 20-μl volumes as follows: denaturation at 95°C for 5 min, followed by 35 cycles of 94°C for 30s, 65°C for 30s, 72°C for 30s. The last cycle was followed by auto-extension 72°C for 7 min with Taq DNA polymerase (Hotstar Taq plus, Qiagen, Valencia, California, USA). The amplified products were analyzed by electrophoresis in 10% polyacrylamide gel (visualized by gel-red).

### Statistical analysis

Statistical analysis was carried out by SPSS20.0 software (SPSS Inc., Chicago, IL). Chi-square test or Fisher’s exact test was used to assess the correlation between the genotype and clinical parametric distributions in colorectal and esophageal cancer patients. The association of *LAPTM4B* gene polymorphism with overall survival (OS) was analyzed using Kaplan-Meier curves and log-rank test. Multivariate analysis determined the potential independent prognostic factors with Cox regression model. All tests of statistical significance were two-sided. *P* < 0.05 was used as statistically significant level.

## Results

### Genotypes of the *LAPTM4B* in colorectal and esophageal cancers

**Fig 1:**
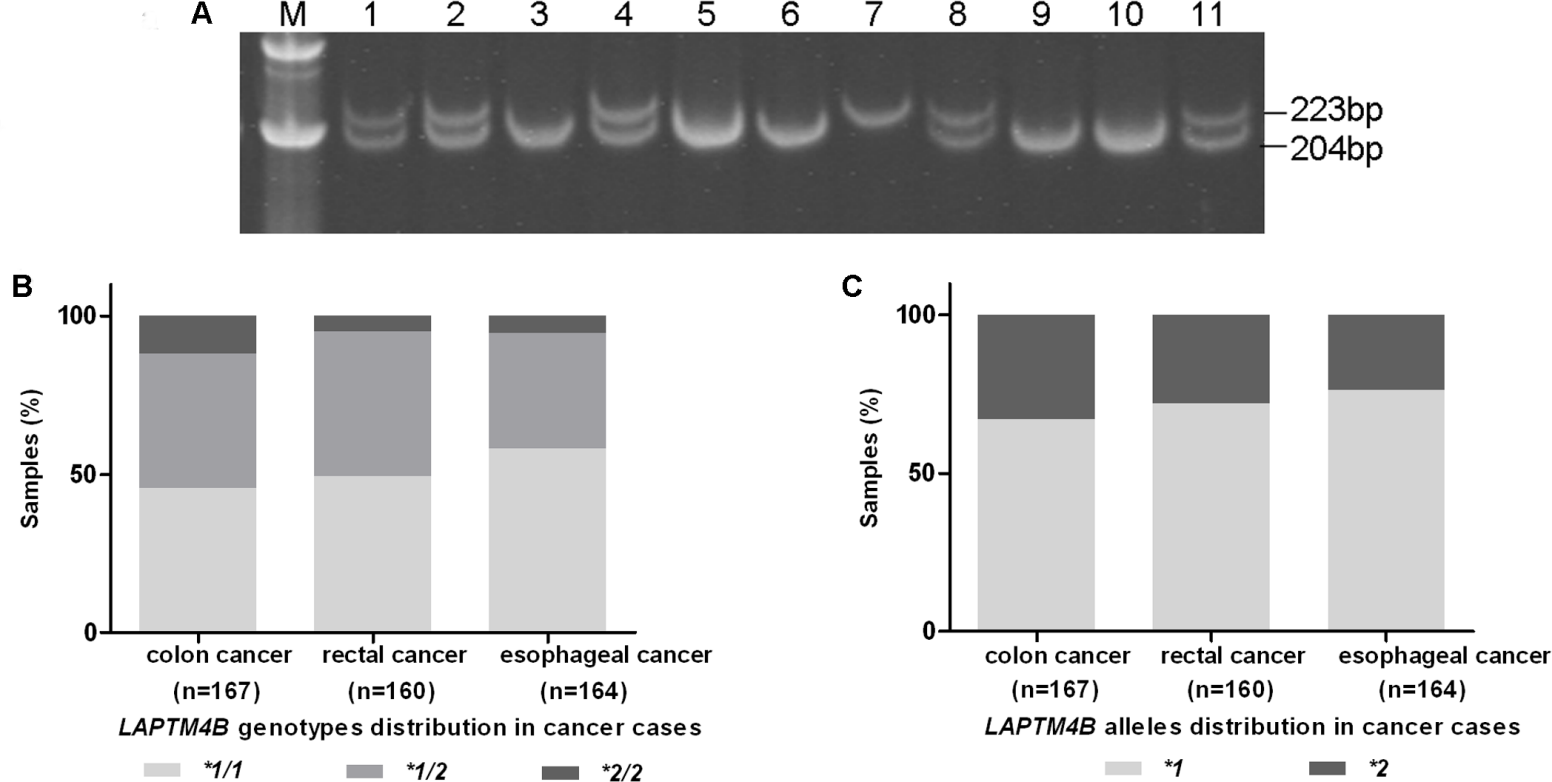
Genotypes of *LAPTM4B* distribution in colorectal and esophageal cancers. (A) Genotypes of *LAPTM4B* were separated in 10% polyacrylamide gel electrophoresis. Lanes 1, 2, 4, 8, 11: LAPTM4B *1/2; lanes 3, 5, 6, 9, 10: LAPTM4B *1/1; lanes 7: LAPTM4B *2/2. (B) Distribution of genotypes of *LAPTM4B* in colorectal and esophageal cancers. *LAPTM4B* genotypes: *1/1, *1/2 and *2/2 frequencies were 44.6%, 42.3% and 13.1%, respectively, in colon cancer; 49.4%, 45.6% and 5.0%, respectively, in rectal cancer; 57.9%, 36.6% and 5.5%, respectively, in esophageal cancer. (C) Distribution of alleles of *LAPTM4B* in colorectal and esophageal cancers. LAPTM4B *1 allele frequency was 65.8% in colon cancer; 72.2% in rectal cancer and 76.2% in esophageal cancer. And LAPTM4B *2 allele frequency was 34.2% in colon cancer; 27.8% in rectal cancer and 23.8% in esophageal cancer.

Three different genotypic *LAPTM4B* polymorphisms: LAPTM4B*1/1, LAPTM4B*2/2 and LAPTM4B*1/2 were shown in Fig 1A. For LAPTM4B*1/1 and LAPTM4B*2/2, we can observe fragments of 204-bp and 223-bp respectively. And both fragments can be observed in LAPTM4B*1/2 heterozygous individual.

The genotypic and allele frequencies of *LAPTM4B* in colorectal and esophageal cancers were depicted in Fig 1B. Among 167 colon cancer cases, the *LAPTM4B* genotypes: *1/1, *1/2 and *2/2 frequencies were 44.6%, 42.3% and 13.1%, respectively. However, the genotype frequencies in 160 rectal cancer cases and 164 esophageal cancer cases were 49.4%, 45.6% and 5.0% vs. 57.9%, 36.6% and 5.5%, respectively (Fig 1B). In colon cancer cases, the LAPTM4B *2 allele frequency was 34.2%, which is different from that in rectal and esophageal cancer cases (27.8% and 23.8%, respectively) (Fig 1C).

### Association between *LAPTM4B* genotypes and clinicopathological parameters in colorectal and esophageal cancers

The distribution of different genotypes of *LAPTM4B* was analyzed in clinicpathological parameters, including age, gender, lymph node metastasis, depth of invasion, distant metastasis, differentiation degree, gross type, TNM stage, location for colon cancer, CEA (carcinoembryonic antigen) for colorectal cancer and recurrence (Table 1).The association of different genotypes of *LAPTM4B* with these clinical variables of colorectal and esophageal cancer patients did not reach statistical significance in our study. LAPTM4B *1/1 was more frequently detected in colon cancer patients with lymph node metastasis and TNM III+IV stages compared with non-lymph node metastasis and TNM I+II stages in total colon cancer (discovery + testing cohorts) (*P* = 0.106, 29.6% vs. 46.6% and *P* = 0.157 31.4% vs. 46.6%, respectively). LAPTM4B *2/2 decreased in recurrent patients compared with non-recurrent ones in total colon cancer patients (*P* = 0.045, 7.4% vs. 20.5%) (Table 1) and in discovery and testing cohort (*P* = 0.203, 7.1% vs. 22.7% and *P* = 0.368, 7.6% vs. 17.2%, respectively) (S1 Table). However, LAPTM4B*1 was more frequently detected in colon cancer patients with moderate and well differentiation in colon discovery cohort (*P* = 0.011) (S1 Table). Such association was not found in colon testing cohort and total colon cancer cases.

**Table 1:** Correlation of Distribution of various genotypes of *LAPTM4B* with clinicopathological parameters in colorectal and esophageal cancer patients.

Variables	Colon cancer				Rectal cancer					Esophagus cancer			
	*1/1	*1/2	*2/2	*P* value	*1/1	*1/2	*2/2	*P* value		*1/1	*1/2	*2/2	*P* value
Age													
≤60	37	31	8	0.528	39	44	3	0.256		46	27	6	0.473
>60	38	40	14		40	29	5			49	33	3	
Gender													
Male	44	41	14	0.886	45	45	4	0.738		80	42	7	0.114
Female	30	30	8		34	28	4			15	18	2	
Lymph node metastasis													
N0	16	27	11	0.106	29	25	4	0.820		33	20	1	0.371
N1+2	34	31	8		35	31	3			61	39	7	
Undetermined	24	13	2		15	17	1			1	1	1	
Depth of invasion													
T1+2	5	5	3	0.691	15	11	1	0.815		26	17	1	0.579
T3+4	46	53	16		52	47	6			70	42	7	
Undetermined	23	13	2		12	15	1			0	1	1	
Distant metastasis													
M0	44	43	17	0.298	54	45	6	0.954		80	47	8	0.149
M1	14	20	3		15	14	2			16	13	0	
Undetermined	16	8	2		10	14	0			0	0	1	
Differentiation													
Poor	12	21	4	0.267	10	12	3	0.196		21	18	2	0.498
Moderate+Well	52	49	17		65	60	4			62	34	8	
Undetermined	10	1	1		4	1	1			13	8	1	
Gross type													
Ulcerative type	31	34	11	0.673	40	40	4	0.152					
Protrude type	10	13	3		8	2	1						
Others	3	8	3		31	31	3						
Undetermined	30	16	4										
TNM stage													
I+II	16	25	10	0.157	26	23	4	0.797		34	20	1	0.363
III+IV	41	37	10		43	35	4			62	40	7	
Undetermined	17	9	2		10	15	0			0	0	1	
Location													
Proximal	24	18	9	0.627									
Distal	33	34	11										
Undetermined	17	19	2										
Recurrence													
No	30	28	15	**0.045**	44	35	5	0.618	80	47	5	0.140	
Yes	44	43	7		36	37	3		16	13	4		
CEA													
Negative	33	31	7	0.984	40	26	3	0.365					
Positive	35	32	8		32	32	5						
Undetermined	6	8	7		8	14	0						

Data was calculated by Chi-square test or Fisher’s exact test.

CEA, carcinoembryonic antigen; *LAPTM4B*, lysosome-associated protein transmembrane 4 beta.

*: Genotype.

### Association of *LAPTM4B* genotypes with overall survival in colorectal and esophageal cancer patients

**Fig 2:**
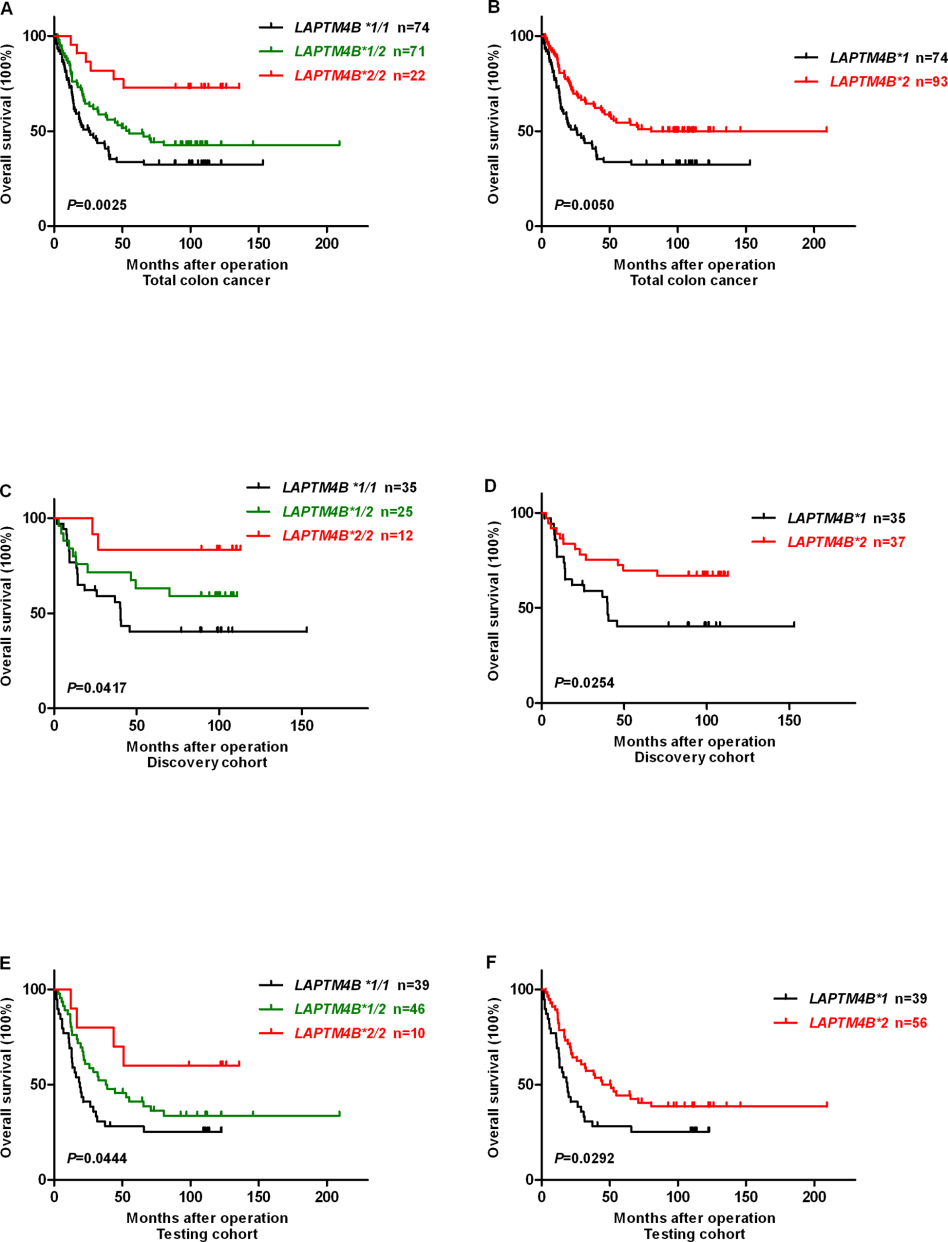
Kaplan-Meier survival curves for survival comparison of colon cancer patients after surgery resection based on *LAPTM4B* genotypes and alleles. Overall survival (OS) after surgery resection based on: (A) *LAPTM4B* genotypes in total colon cancer (*P* = 0.025). (B) *LAPTM4B* alleles in total colon cancer (*P* = 0.0050). (C and D) *LAPTM4B* genotypes and alleles in colon discovery cohort (*P* = 0.0417 and 0.0254), respectively. (E and F) *LAPTM4B* genotypes and alleles in colon testing cohort (*P* = 0.0444 and 0.0292), respectively.

**Fig 3:**
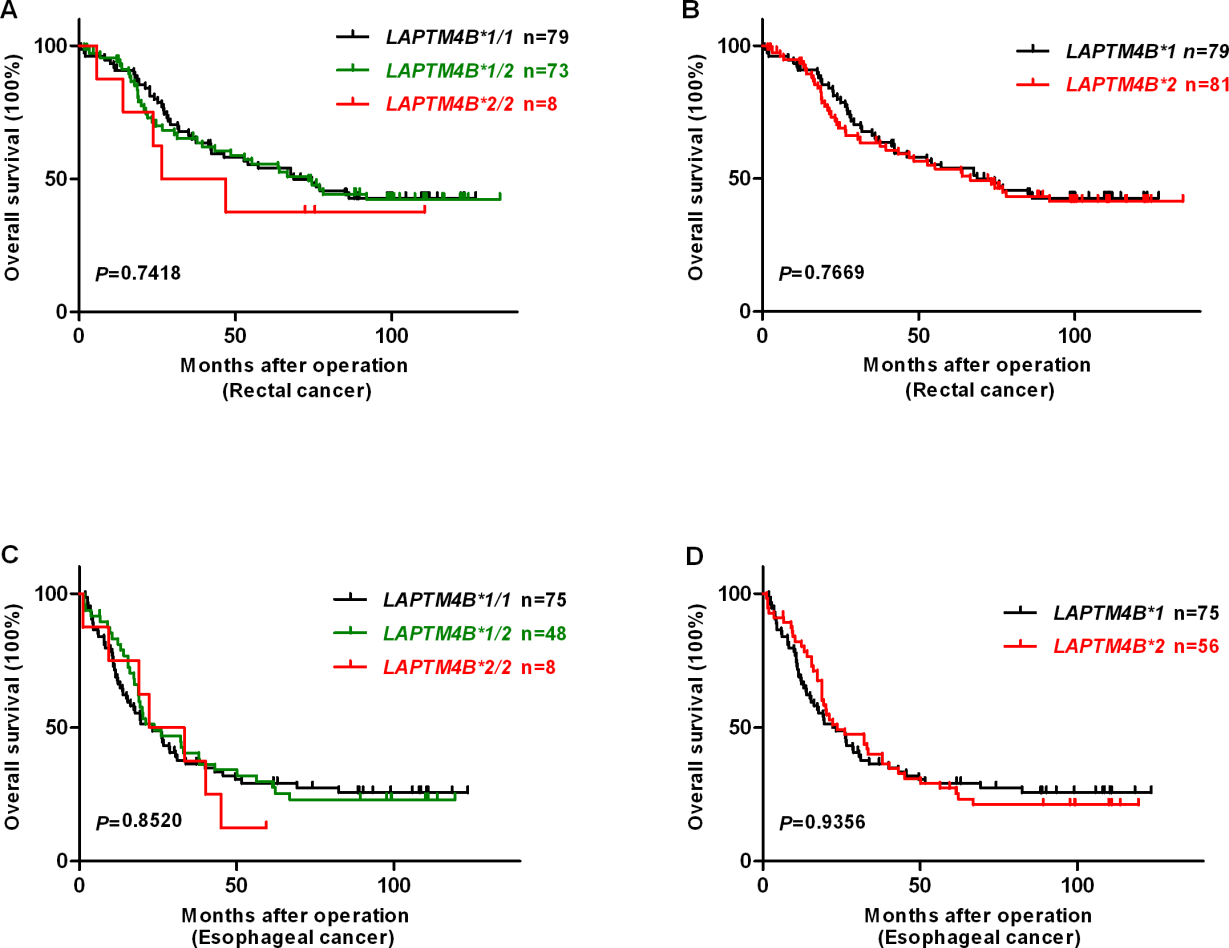
Kaplan-Meier survival curves for survival comparison of rectal and esophageal cancer patients undergone surgery resection based on *LAPTM4B* genotypes and alleles. Overall survival (OS) after surgery resection based on: (A and B) *LAPTM4B* genotypes and alleles in rectal cancer (*P* = 0.7418 and 0.7669), respectively. (C and D) *LAPTM4B* genotypes and alleles in esophageal cancer (*P* = 0.8520 and 0.9356), respectively.

Kaplan-Meier survival analysis and log-rank test indicated that colon cancer patients with LAPTM4B *1/1 genotype showed a shorter overall survival (OS) when compared with those with LAPTM4B *1/2 and LAPTM4B *2/2 genotypes (OS rate 33.8% vs. 43.7% and 72.7%, *P* = 0.0025) (Fig 2A). Moreover, patients with LAPTM4B *1 allele had a poorer prognosis than LAPTM4B *2 allele in total colon cancer cases (OS rate 33.8% vs. 50.5%, *P* = 0.0050) (Fig 2B). The same tendency of LAPTM4B *1/1 and LAPTM4B *1 was observed in the colon discovery and testing cohorts of colon cancer (*P* = 0.0417 and 0.0444, *P* = 0.0254 and 0.0292, respectively) (Fig 2C–2F). However, we did not find a marked relation between *LAPTM4B* genotypes or alleles and OS for rectal and esophageal cancer cases (*P* = 0.7418 and 0.8520 for various genotypes vs. *P* = 0.7669 and 0.9356 for different alleles, respectively) (Fig 3A–3D).

### *LAPTM4B* genotype was an independent prognostic marker in patients with colon cancer, but not for rectal and esophageal cancer patients

The univariate Cox’s model for OS of colorectal and esophageal cancer patients displayed that *LAPTM4B* genotype was one of the prognostic factors in total colon cancer patients (*P* = 0.006; HR: 0.565, 95% CI: 0.377–0.846), not in rectal and esophageal cancer patients (*P* = 0.692 and 0.958, respectively) (Table 2).

**Table 2:** Univariate analysis of the prognostic factors in colorectal and esophageal cancer patients by Log-rank test.

Variables	Colon cancer			Rectal cancer			Esophageal cancer		
	Univariate analysis			Univariate analysis			Univariate analysis		
	HR	95% CI	*P* value	HR	95% CI	*P* value	HR	95% CI	*P* value
Age									
≤60	1.468	0.969–2.222	0.070	1.069	0.702–1.628	0.757	1.415	0.948–2.112	0.090
>60									
Gender									
Male	0.782	0.515–1.186	0.247	1.196	0.783–1.827	0.409	0.584	0.336–1.015	0.057
Female									
Lymph node metastasis									
N0	4.385	2.375–8.096	**<0.001**	1.838	1.115–3.031	**0.017**	1.628	1.041–2.548	**0.033**
N1+2									
Depth of invasion									
T1+2	1.841	0.669–5.076	0.237	1.903	0.997–3.630	0.051	1.900	1.110–3.255	**0.019**
T3+4									
Distant metastasis									
M0	6.687	4.134–10.815	**<0.001**	4.990	3.012–8.266	**<0.001**	2.306	1.390–3.825	**0.001**
M1									
Differentiation									
Poor	1.116	0.679–1.836	0.665	0.779	0.438–1.384	0.394	1.348	0.825–2.201	0.233
Moderate+Well									
Gross type									
Ulcerative type	1.035	0.717–1.496	0.853	0.427	0.133–1.374	0.154			
Protrude type									
Others									
TNM stage									
I+II	6.434	3.290–12.583	**<0.001**	2.415	1.428–4.084	**0.001**	1.627	1.048–2.526	**0.030**
III+IV									
Location									
Proximal	1.194	0.726–1.692	0.485						
Distal									
Recurrence									
No	5.943	3.533–9.999	**<0.001**	4.073	2.557–6.488	**<0.001**	1.882	1.219–2.904	**0.004**
Yes									
CEA									
Negative	1.651	1.072–2.545	**0.023**	1.865	1.184–2.936	**0.007**			
Positive									
*LAPTM4B* genotype									
*1/1	0.565	0.377–0.846	**0.006**	1.089	0.715–1.658	0.692	0.989	0.665–1.472	0.958
*1/2+ *2/2									

Data was calculated by Log-rank test. HR, hazard ratio; CI, confidence interval.

CEA, carcinoembryonic antigen; *LAPTM4B*, lysosome-associated protein transmembrane 4 beta.

*: Genotype.

Furthermore, *LAPTM4B* genotype was a novel independent prognostic factor of OS for colon cancer (*P* = 0.004, HR = 0.432, 95% CI: 0.243–0.768), even in the colon discovery cohorts (*P* = 0.007, S2 Table), but not for rectal and esophageal cancer patients (*P* = 0.791, HR = 1.073, 95% CI: 0.638–1.804 vs. *P* = 0.998, HR = 1.000, 95% CI: 0.663–1.530, respectively). Depth of invasion, distant metastasis and recurrence were also independent prognostic factors for colon cancer (*P* = 0.014, *P*<0.001 and *P*<0.001, respectively). For rectal cancer patients, distant metastasis and recurrence were also independent prognostic factors (*P* = 0.021 and *P*<0.001, respectively). In addition, distant metastasis (*P* = 0.025) was also an independent prognosis factor in esophageal cancer patients (Table 3).

**Table 3:** Multivariate analysis of the prognostic factors in colorectal and esophageal cancer patients by Cox proportional hazard regression model.

Variables	Colon cancer			Rectal cancer			Esophageal cancer		
	Multivariate analysis			Multivariate analysis			Multivariate analysis		
	HR	95% CI	*P* value	HR	95% CI	*P* value	HR	95% CI	*P* value
Age									
≤60 vs >60	1.019	0.565–1.838	0.949	1.211	0.712–2.062	0.480	1.313	0.852–2.022	1.313
Gender									
Male vs Female	0.864	0.498–1.500	0.603	1.225	0.705–2.129	0.471	0.765	0.408–1.434	0.765
Depth of invasion									
T1+2 vs T3+4	4.240	1.275–14.102	**0.018**	1.494	0.689–3.242	0.309	1.528	0.848–2.751	0.158
Lymph node metastasis									
N0 vs N1+3	0.950	0.614–1.470	**0.819**	1.186	0.654–2.148	0.575	1.200	0.723–1.992	0.480
Distant metastasis									
M0 vs. M1	4.517	2.281–8.945	**<0.001**	2.173	1.122–4.208	**0.021**	1.988	1.089–3.629	0.025
Recurrence									
No vs. Yes	6.898	3.061–15.543	**<0.001**	3.748	1.896–7.411	**<0.001**	1.577	0.980–2.537	0.061
CEA									
Negative vs. Positive	0.828	0.466–1.471	0.519	1.539	0.895–2.648	0.120			
*LAPTM4B* genotype									
*1/1 vs *1/2+*2/2	0.432	0.243–0.768	**0.004**	1.073	0.638–1.804	0.791	1.000	0.663–1.530	0.998

Data was calculated by Cox regression test. HR, hazard ratio; CI, confidence interval.

CEA, carcinoembryonic antigen; *LAPTM4B*, lysosome-associated protein transmembrane 4 beta.

*: Genotype.

## Discussion

Previous studies have demonstrated that LAPTM4B can play critical roles in various solid tumors, including proliferation, migration, invasion, apoptosis and angiogenesis [12, 27–29]. It also motivated multidrug resistance through promoting drug efflux by interacting with P-gp and activating PI3K/AKT signaling pathway [30]. In addition, new evidence has also revealed that LAPTM4B can participate in the autophagy initiation through binding with inactive epidermal growth factor receptor (EGFR) [31, 32].

In the present study, we revealed an independent prognostic role of *LAPTM4B* gene polymorphism in colon cancer patients who received surgical resection, but not in rectal and esophageal cancers. In our study, the LAPTM4B *2 allele frequency rate are 33.3%, 27.8% and 23.8%, nearly the same as previous report in colon, rectal and esophageal cancers [20], respectively. For the clinicopathological parameters, *LAPTM4B* genotype was correlated with recurrence in total colon cancer, especially for LAPTM4B *2/2 which decreased in recurrent colon cancer patients. There was not a relationship between *LAPTM4B* genotype and other clinical factors in colorectal and esophageal cancer patients. However, LAPTM4B*1 was more frequently detected in colon cancer patients with moderate and well differentiation in colon discovery cohort. This phenomenon might be caused by incomplete clinicopathological parameters in the present work. Furthermore, LAPTM4B *1/1 tended to be frequently detected in patients with lymph node metastasis and TNM III+IV stages in total colon cancer cases.

Patients with LAPTM4B *1 (genotypes *1/1) had a significantly poorer overall survival when compared with LAPTM4B *2 (genotypes *1/2 or *2/2) patients in colon cancer (discovery and testing cohorts), but not in rectal and esophageal cancers. This is the first time demonstrating the LAPTM4B *1 allele as a poor prognostic indicator. The association of LAPTM4B *2 allele with colon cancer prognosis is not consistent with recent reports including in hepatocellular [23], ovary [27], lung [23], breast cancer [25] and etc. In hepatocellular carcinoma, Yang et al indicated that LAPTM4B*2 was correlated with tumor recurrence, poor histopathological differentiation and also an independent prognostic factor. Previous studies indicated that the 19-bp difference in 5’ untranslated region of the first exon of the *LAPTM4B* gene can alter the ORF, resulting in two different protein isoforms: LAPTM4B-35 and -40 [14]. It might suggest that the 19-bp sequence plays an important role in transcriptional regulation or new isoform produced by LAPTM4B*2 may influence physiological activity and function of cancer cells.

Whereas in our study, LAPTM4B *1 allele shows a significant correlation with overall survival of colon cancer patients, but not in rectal and esophageal cancer patients. One explanation might be: the diverse expression patterns or isoforms of LAPTM4B in epithelial cells might demonstrate the difference of LAPTM4B genotype in prognosis in colon cancer vs rectal and esophageal cancers; the other explanation might be: the 19-bp sequence may play an important role in transcriptional regulation such as binding with the transcription factors or non-coding linker RNA in different cancers. Furthermore, in patients with gastric cancer, we have found that even though *LAPTM4B* genotype was correlated with susceptibility of gastric cancer, this polymorphism did not correlate with prognosis (data unpublished). The phenomenon illustrated the tumor heterogenicity between *LAPTM4B* genotype and its function, which discriminates with that in hepatocellular carcinoma, breast cancer and etc.

LAPTM4B was obviously up-regulated in various types of cancers [6]. Its overexpression might be caused by gene amplification and transcriptional up-regulation. However, the specific reason remains unknown. LAPTM4B has two different protein isoforms: LAPTM4B-24 (226 aa) and LAPTM4B-35 (317 aa). Li et al. has indicated that LAPTM4B-35 isoform can activate PI3K/Akt to participate multidrug resistance of cancer cells and anti-apoptosis [30]. Previous studies have proved that LAPTM4B-35 and -24 have different expression status and different roles in tissues and various cell lines of hepatocellular carcinoma [29, 33, 34]. Their balance may affect malignant transformation. However, a recent report has shown that LAPTM4B-24 isoform can stimulate mTORC1 via V-ATPase by influx of leu through binding with LAT1-4F2hc to lysosomes [35]. LAPTM4B-24 can also promote cell growth and proliferation [35].

As a result, the different LAPTM4B isoforms may play diverse functions in LAPTM4B *1 patients when compared with LAPTM4B *2 patients with colon cancer. Our findings on *LAPTM4B* alleles in colon cancer provide additional evidence that different *LAPTM4B* isoforms might play various roles, that is, LAPTM4B-35 can activate PI3K/Akt pathway and LAPTM4B -24 can activate mTORC1 pathway. Different isoform pattern might induce the function of 19 bp sequence in various cancers. Further studies should be carried out to elucidate this phenomenon. However, *LAPTM4B* genotype will be a useful biomarker for colon cancer patients when considering curative surgical resection.

## Supporting Information

S1 Table Correlation of distribution of various genotypes of LAPTM4B with clinicopathological parameters in discovery and testing cohorts of colon cancer patients. (DOCX)

S2 Table Multivariate analysis of the prognostic factors in discovery and testing cohorts of colon cancer patients by Cox proportional hazard regression model. (DOCX)
