# Supplementary material for: Relationship between LAPTM4B Gene Polymorphism and Prognosis of Patients following Tumor Resection for Colorectal and Esophageal Cancers
Source: PLoS One. 2016 Jul 8;11(7):e0158715. doi: 10.1371/journal.pone.0158715 (PMC4938575; doi:10.1371/journal.pone.0158715)
Supplement: S1 Table — (DOCX) [file pone.0158715.s001.docx]

**S1 Table: Correlation of distribution of various genotypes of LAPTM4B with clinicopathological parameters in discovery and testing cohorts of colon cancer patients**

| Variables |  | Discovery cohort | | | |  | Testing cohort | | | |
| --- | --- | --- | --- | --- | --- | --- | --- | --- | --- | --- |
|  |  | **1/1* | **1/2* | **2/2* | *P* value |  | **1/1* | **1/2* | **2/2* | *P* value |
| Age |  |  |  |  |  |  |  |  |  |  |
| ≤60 |  | 21 | 12 | 3 | 0.109 |  | 15 | 19 | 5 | 0.804 |
| >60 |  | 14 | 13 | 9 |  |  | 24 | 27 | 5 |  |
| Gender |  |  |  |  |  |  |  |  |  |  |
| Male |  | 19 | 15 | 7 | 0.902 |  | 25 | 26 | 7 | 0.639 |
| Female |  | 16 | 10 | 5 |  |  | 14 | 20 | 3 |  |
| Lymph node metastasis | | |  |  |  |  |  |  |  |  |
| N0 |  | 9 | 9 | 8 | 0.097 |  | 7 | 18 | 3 | 0.270 |
| N1+2 |  | 14 | 9 | 2 |  |  | 20 | 22 | 6 |  |
| Undetermined |  | 12 | 7 | 2 |  |  | 12 | 6 | 1 |  |
| Depth of invasion | | |  |  |  |  |  |  |  |  |
| T1+2 |  | 2 | 2 | 2 | 0.674 |  | 3 | 3 | 1 | 0.880 |
| T3+4 |  | 21 | 16 | 8 |  |  | 25 | 37 | 8 |  |
| Undetermined |  | 12 | 7 | 2 |  |  | 11 | 6 | 1 |  |
| Distant metastasis | | |  |  |  |  |  |  |  |  |
| M0 |  | 20 | 17 | 9 | 0.941 |  | 24 | 26 | 8 | 0.220 |
| M1 |  | 6 | 5 | 2 |  |  | 8 | 15 | 1 |  |
| Undetermined |  | 9 | 3 | 1 |  |  | 7 | 5 | 1 |  |
| Differentiation | |  |  |  |  |  |  |  |  |  |
| Poor |  | 1 | 8 | 3 | **0.011** |  | 11 | 13 | 1 | 0.443 |
| Moderate+Well |  | 27 | 16 | 9 |  |  | 25 | 33 | 8 |  |
| Undetermined |  | 7 | 1 | 0 |  |  | 3 | 0 | 1 |  |
| Gross type |  |  |  |  |  |  |  |  |  |  |
| Ulcerative type |  | 16 | 12 | 5 | 0.878 |  | 15 | 22 | 6 | 0.104 |
| Protrude type |  | 5 | 6 | 2 |  |  | 5 | 7 | 1 |  |
| Others |  | 11 | 6 | 5 |  |  | 0 | 7 | 2 |  |
| Undetermined |  |  |  |  |  |  | 19 | 10 | 1 |  |
| TNM stage |  |  |  |  |  |  |  |  |  |  |
| I+II |  | 9 | 9 | 7 | 0.230 |  | 7 | 16 | 3 | 0.369 |
| III+IV |  | 18 | 12 | 4 |  |  | 23 | 25 | 6 |  |
| Undetermined |  | 8 | 4 | 1 |  |  | 9 | 5 | 1 |  |
| Location |  |  |  |  |  |  |  |  |  |  |
| Proximal |  | 19 | 8 | 5 | 0.226 |  | 5 | 10 | 4 | 0.320 |
| Distal |  | 16 | 17 | 7 |  |  | 17 | 17 | 4 |  |
| Undetermined |  |  |  |  |  |  | 17 | 19 | 2 |  |
| Recurrence |  |  |  |  |  |  |  |  |  |  |
| No |  | 19 | 15 | 10 | 0.203 |  | 11 | 13 | 5 | 0.368 |
| Yes |  | 16 | 10 | 2 |  |  | 28 | 33 | 5 |  |
| CEA |  |  |  |  |  |  |  |  |  |  |
| Negative |  | 19 | 8 | 4 | 0.250 |  | 14 | 23 | 3 | 0.283 |
| Positive |  | 12 | 13 | 3 |  |  | 23 | 19 | 5 |  |
| Undetermined |  | 4 | 4 | 5 |  |  | 2 | 4 | 2 |  |

Data was calculated by Chi-square test or Fisher’s exact test.

CEA, carcinoembryonic antigen.

LAPTM4B, lysosome-associated protein transmembrane 4 beta.

*: Genotype.
