# Supplementary material for: Relationship between LAPTM4B Gene Polymorphism and Prognosis of Patients following Tumor Resection for Colorectal and Esophageal Cancers
Source: PLoS One. 2016 Jul 8;11(7):e0158715. doi: 10.1371/journal.pone.0158715 (PMC4938575; doi:10.1371/journal.pone.0158715)
Supplement: S2 Table — (DOCX) [file pone.0158715.s002.docx]

**S2 Table: Multivariate analysis of the prognostic factors in discovery and testing cohorts of colon cancer patients by Cox proportional hazard regression model.**

| Variables |  | Discovery cohort | | |  | Testing cohort | | |
| --- | --- | --- | --- | --- | --- | --- | --- | --- |
|  |  | Multivariate analysis | | |  | Multivariate analysis | | |
|  |  | **HR** | **95% CI** | *P* value |  | **HR** | **95% CI** | *P* value |
| Age |  |  |  |  |  |  |  |  |
| ≤60 vs >60 |  | 0.279 | 0.040-1.919 | 0.194 |  | 0.514 | 0.643-2.417 | 1.247 |
| Gender |  |  |  |  |  |  |  |  |
| Male vs Female |  | 0.264 | 0.031-2.271 | 0.225 |  | 0.580 | 0.307-1.097 | 0.094 |
| Depth of invasion |  |  |  |  |  |  |  |  |
| T1+2 vs T3+4 |  | 0.315 | 0.017-6.001 | 0.443 |  | 8.997 | 1.966-41.171 | **0.005** |
| Lymph node metastasis |  |  |  |  |  |  |  |  |
| N0 vs N1+3 |  | 24.219 | 1.512-388.002 | 0.024 |  | 2.937 | 1.209-7.133 | **0.017** |
| Distant metastasis |  |  |  |  |  |  |  |  |
| M0 vs. M1 |  | 59.784 | 2.944-1214.053 | **0.008** |  | 2.746 | 1.284-5.870 | **0.009** |
| Recurrence |  |  |  |  |  |  |  |  |
| No vs. Yes |  | 0.761 | 0.061-9.465 | 0.831 |  | 7.856 | 2.609-23.651 | **<0.001** |
| CEA |  |  |  |  |  |  |  |  |
| Negative vs. Positive |  | 0.595 | 0.104-3.403 | 0.560 |  | 1.117 | 0.568-2.197 | 0.749 |
| *LAPTM4B* genotype |  |  |  |  |  |  |  |  |
| **1/1* vs **1/2*+**2/2* |  | 0.021 | 0.001-0.342 | **0.007** |  | 0.595 | 0.304-1.163 | 0.129 |

Data was calculated by Cox regression test. HR, hazard ratio; CI, confidence interval.

CEA, carcinoembryonic antigen.

LAPTM4B, lysosome-associated protein transmembrane 4 beta.

*: Genotype.
